# The Impact of Controlling Nutritional Status (CONUT) score on functional prognosis in hospitalized elderly patients with acute osteoporotic vertebral fractures

**DOI:** 10.1186/s12877-022-03708-x

**Published:** 2022-12-28

**Authors:** Tetsuto Yamaura, Fumihiro Arizumi, Keishi Maruo, Kazuya Kishima, Norichika Yoshie, Tomoyuki Kusukawa, Fumiaki Imamura, Toshiya Tachibana

**Affiliations:** 1 Department of Orthopaedic Surgery, JCHO Osaka Minato Central Hospital, Osaka, Japan; 2 Department of Orthopaedic Surgery, Hyogo Medical University, 1-1 mukogawa-cho, Nishinomiya, Hyogo Japan

**Keywords:** Controlling Nutritional Status score, nutritional status, osteoporotic vertebral fractures

## Abstract

**Background:**

Nutritional status, which is associated with osteoporosis and muscle weakness is considered an important factor in the management of acute osteoporotic vertebral fracture (AOVF). However, few reports have investigated the nutritional status of hospitalized patients with AOVF and the impact of malnutrition on their functional prognosis. This study aimed to evaluate the nutritional status of hospitalized elderly patients with AOVF using the Controlling Nutritional Status (CONUT) score and to determine the usefulness of the CONUT score in predicting their functional prognosis.

**Methods:**

The CONUT score on admission was retrospectively calculated for 134 hospitalized elderly patients (mean age 83 ± 7.6 years, 66% female) with AOVF who received conservative treatment between 2017 and 2020. Functional outcome was assessed by comparing ambulatory ability before the onset of AOVF and upon discharge. Patients were divided into two groups: CONUT-high ( ≥ 4) and CONUT-low ( ≤ 3), according to receiver operating characteristic (ROC) analysis to predict decline in ambulatory ability upon discharge. Logistic regression analysis was performed to obtain odds ratios (OR) and 95% confidence intervals (CI) of the relationships between the nutritional status and ambulatory ability. The discriminative power of the CONUT score was then compared with other nutritional assessment tools such as the Geriatric Nutritional Risk Index (GNRI) and prognostic nutritional index (PNI) by ROC analysis.

**Results:**

81% of hospitalized patients with an AOVF were malnourished at the time of admission. The CONUT-high group had a significantly higher rate of decline in ambulatory ability (*P *< 0.001) than the CONUT-low group. Logistic regression analysis revealed the CONUT score ( ≥ 4) as an independent risk factor for a decline in ambulatory ability (OR 3.44, 95% CI 1.61–7.37, *P = *0.0014). ROC analysis showed that the area under the curve (AUC) for the CONUT score (AUC = 0.724) was significantly greater than that for the GNRI (AUC = 0.624, *P* = 0.021) and PNI (AUC = 0.636, *P *= 0.0008).

**Conclusions:**

This study showed that 81% of hospitalized elderly patients with AOVFs were malnourished and that the CONUT score was a useful predictive factor of functional prognosis.

## Introduction

Vertebral fractures are recognized as the most common osteoporotic fractures in elderly people, and the number of patients with osteoporotic vertebral fractures (OVF) is increasing with the aging population. Fragility fractures, including OVFs, have been reported to be associated with frailty, sarcopenia, dysphagia, malnutrition and various complications such as COPD, especially in the elderly. Thus, fragility fractures in elderly patients require comprehensive intervention [1–3]. Among fragility fractures, acute OVF (AOVF) causes pain, disability, a decline of activities of daily living (ADL), reduced Quality of Life (QoL) and an increased rate of mortality [4, 5]. Therefore, there is an increasing interest in the management for the treatment and prevention of AOVF. In addition, patients with AOVF which is common in the elderly, require not only treatment for OVF, but also various interventions to prevent complications, frailty, sarcopenia and malnutrition. Nutritional status has been reported to be associated with osteoporosis, sarcopenia, and muscle loss [6, 7], which have been recognized as risk factors for the development of AOVF and poor prognostic factors for the clinical outcome of elderly patients with AOVFs [8, 9]. We hypothesize that nutritional status is an important factor affecting clinical outcomes in the management of patients with OVF. However, few reports have investigated the nutritional status of patients with AOVFs and the impact of malnutrition on functional prognosis.

The Controlling Nutritional Status (CONUT) score is a tool that is used to screen and identify hospitalized patients with malnutrition [10]. The score is based on the patient’s serum albumin level, total cholesterol, and total lymphocyte count (TLC) and only requires common laboratory tests. CONUT score is one of the Objective Data Assessment (ODA) tools that consists of objective data provided from various analyses and allows examiner-independent evaluation for nutritional status [11]. Gonzalez et al showed that the CONUT score is highly correlated with Subjective Global Assessment (SGA), an established tool renowned for its reliable [12]. In addition to CONUT score, the Geriatric Nutritional Risk Index (GNRI) and prognostic nutritional index (PNI) were well-known as ODA tools [13, 14]. These tools were well considered in relation to the prognosis of cancer patients [15–17]. Recently there have been a growing number of reports on these tools in many clinical areas, and several studies have compared the usefulness of these tools [18, 19]. However, few reports have demonstrated the usefulness of these tools in predicting functional prognosis of AOVF patients. The aim of this study was to evaluate the nutritional status of hospitalized elderly patients with AOVF using the CONUT score, and to determine the usefulness of the CONUT score in predicting their short-term functional prognoses and compare its functional prognostic usefulness with the GNRI and PNI.

## Materials and methods

### Study patients

We retrospectively reviewed clinical data of 192 patients who were hospitalized for conservative treatment of AOVF between January 2017 and March 2020 at a single center. The study was reviewed by Ethics Committee of Hyogo Medical University (Nishinomiya City, Japan) and approved on 20 June 2022 (ID number: 4112). All our experiments were performed in accordance with the Declaration of Helsinki, relevant guidelines and regulations. All patients signed an informed consent agreement. The inclusion criteria were as follows: patients aged 65 years or older; AOVF diagnosed by magnetic resonance imaging (MRI); fragility fracture without trauma or resulting from low-energy trauma, such as a fall from standing or sitting; and admission for severe lower back pain. Exclusion criteria included: required surgery due to a neurological deficit or nonunion following conservative treatment for OVF; pathological vertebral fracture; previous spinal surgery; concomitant non-spinal associated injury; a lack of radiographic and blood sampling data; and death during hospitalization. Anteroposterior and lateral radiographs and a 1.5T MRI were performed, and blood samples were taken on the day of admission or the following day. AOVF was diagnosed by MRI, which showed a hypointense area on T1-weighted imaging (T1WI) and hyperintense lesions on short T1 inversion recovery (STIR) imaging. The indication for admission was notable disability due to severe lower back pain. The conservative treatment protocol included bed rest for the first week, physical therapy, implementation of a soft or hard brace, evaluation for osteoporosis, and drug treatment if indicated. The type of brace was determined by the patient or physician based on age, ADL, compliance, and medical comorbidities.

### Measurements

All clinicopathological data were collected through the electronic medical record system. Demographic and laboratory measurements included age, sex, weight, body mass index (BMI), comorbidities, osteoporosis treatment, serum albumin, total cholesterol, TLC, and hemoglobin (Hb). Radiographic measurements included level of AOVFs, the presence of multiple AOVFs and prevalent OVFs, lumbar lordosis (LL), and bone mineral density (BMD) by Dual-energy X-ray absorptiometry (DXA). AOVF was defined as a vertebral signal intensity change on T1WI or STIR, and prevalent OVF as a 15% height reduction of the front edge of the vertebral body with no signal intensity change on T1WI or STIR [20]. Two of the authors—spine surgeons with 7 and 16 years of experience—diagnosed the presence of AOVFs and prevalent OVFs.

### The CONUT score and other nutritional assessment tools

The CONUT score was calculated based on serum albumin level, total cholesterol concentration, and TLC. The CONUT score is a sum of the scores based on the serum albumin (0, 2, 4 ,6), total cholesterol concentration, and TLC (0, 1, 2, 3, for each), and classified as normal, light, moderate, or severe for scores of 0-1, 2-4, 5-8 and 9-12, respectively (Table 1) [10]. GNRI and PNI were calculated as below: GNRI = 14.89 × serum albumin (g/dl) + {41.7 × (current/ideal body weight)}; PNI = 10 × serum albumin (g/dl) + 0.005 × TLC (/μl).

**Table 1:** Controlling Nutritional Status (CONUT) score calculation

		Undernutrition Degree		
Parameter	Normal	Light	Moderate	Severe
Serum Albumin (g/dl)	3.5–4.5	3.0–3.49	2.5–2.9	$<$ 2.5
Score	0	2	4	6
Total lymphocytes (/mm^3^)	$>$ 1600	1200–1599	800–1199	$<$ 800
Score	0	1	2	3
Cholesterol (mg/dl)	$>$ 180	140–180	100–139	$<$ 100
Score	0	1	2	3
Screening total score	0–1	2–4	5–8	9–12

### Outcome measurements

The primary outcome measure was level of ambulatory ability. Ambulatory ability was categorized into 5 levels: level 1 (bedridden), level 2 (wheelchair use), level 3 (walker use), level 4 (cane use), and level 5 (independent gait). Levels of ambulatory ability were evaluated before the onset of AOVF and upon discharge. Patient’s ambulatory ability just before the onset of pain due to AOVF was interviewed from patients with AOVF or their family members at the first examination. A decline in ambulatory ability was defined as a decrease of one or more levels. Secondary outcome measures were complications during hospitalization, length of hospital stay, and rate of discharge to nursing home.

### Statistical analysis

The statistical software JMP® 15 (SAS Institute Inc., Cary, NC, USA) was used for data analysis. *P* < 0.05 was considered statistically significant. Continuous variables were represented as mean and standard deviation (SD) or median (25th-75th quartile), and the categorical variables were presented as percentages and numerical values. Student’s t-test was used to analyze normally distributed variables, and the Mann-Whitney U test was used to analyze non-normal distributions. The chi-squared test or Fisher’s exact test was used to analyze the categorical variables. The relationship between CONUT score and ambulatory ability was examined using logistic regression models. Univariate analysis was performed to determine associated factors. Variables with *P* ≤ 0.1 in univariate analysis were used as covariates in the multivariate logistic regression analysis. Adjusted odds ratio (OR) with 95% confidence intervals (CI) are presented along with their respective P value. The receiver operating characteristic (ROC) analysis were performed to demonstrate the sensitivity and specificity of CONUT score, GNRI and PNI, and their cut-off values for predicting the decline in ambulatory ability. The DeLong test was used to compare the area under the curve (AUC) of each nutritional assessment tool (CONUT, GNRI, PNI). A priori power analysis and sample size calculation were not conducted. However, a post hoc power analysis with α = 0.05 for the primary outcome comparison between CONUT low and high group was conducted, using Stata 17.0 software (Stata Corporation, College Station, TX, USA).

## Results

**Fig. 1:**
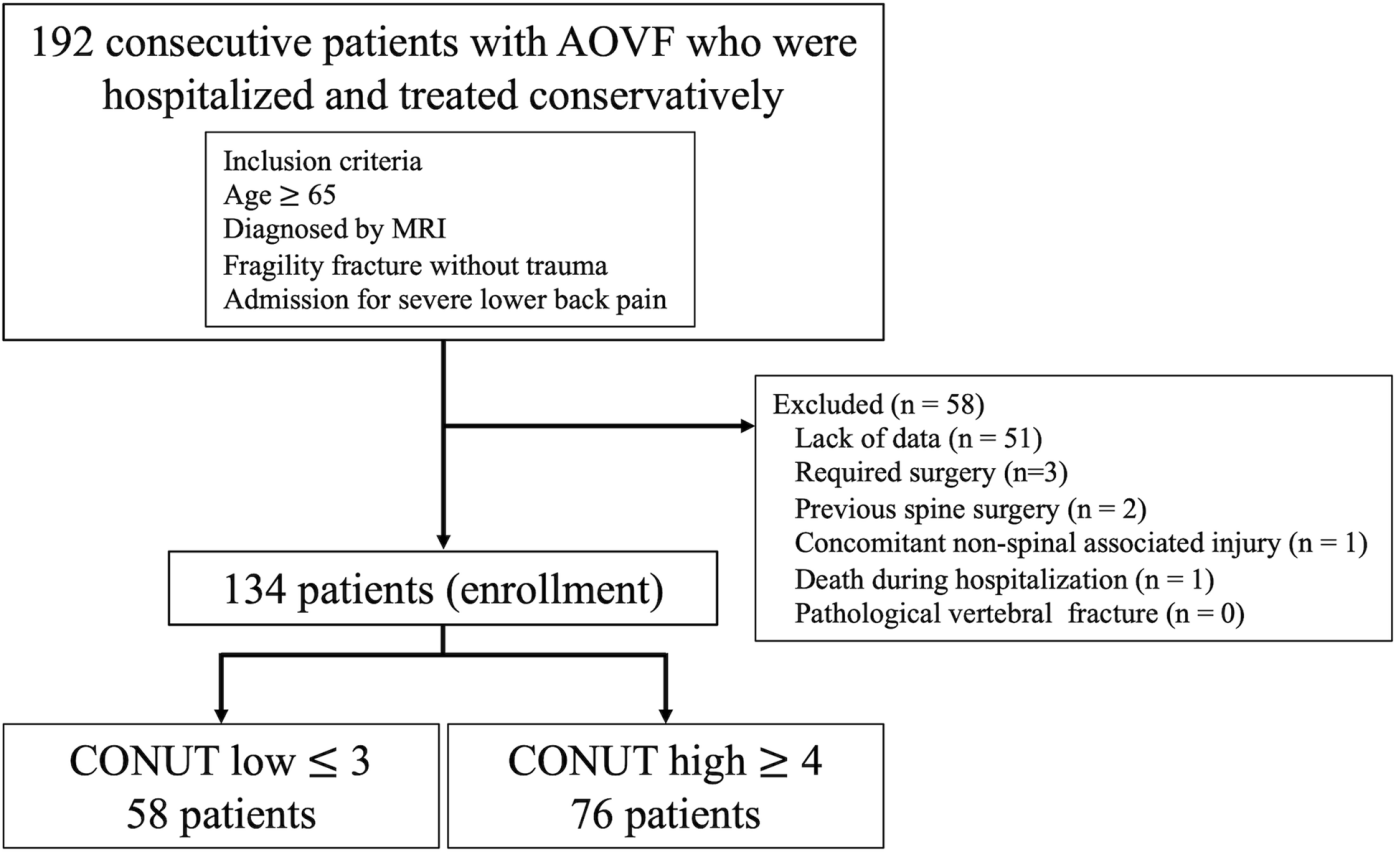
Flow diagram of patients evaluated in the present study. AOVF, acute osteoporotic vertebral fracture; MRI, magnetic resonance imaging; CONUT, Controlling Nutritional Status

**Fig. 2:**
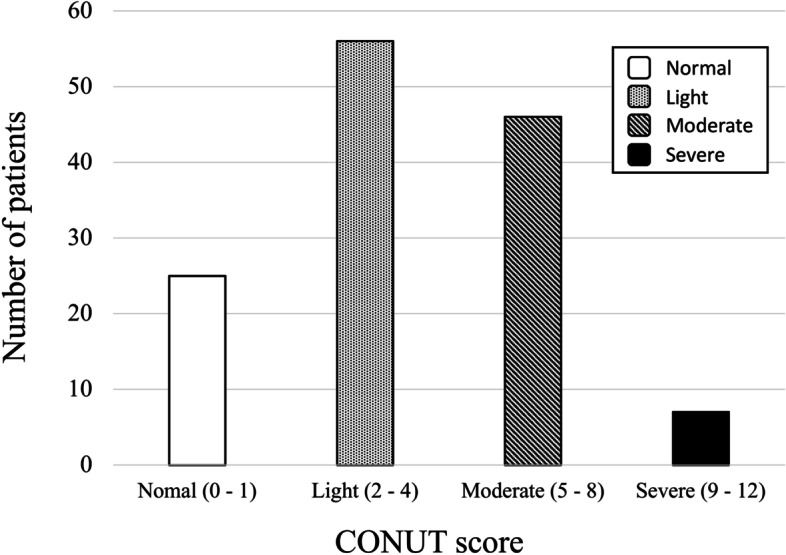
Distribution and classification of the CONUT score. CONUT, Controlling Nutritional Status

Figure 1 shown flow diagram of patients evaluated in the present study. Of the 192 patients that met the inclusion criteria in the present, 58 patients were removed due to the exclusion criteria for a total of 134 patients enrolled. Patient demographics and clinical characteristics are shown in Table 2. 89 patients were female, and the mean age was 83.5 ± 7.6 years. Of those patients who were enrolled, 112 (84%) had medical comorbidities including hypertension, diabetes, cardiovascular disease, dementia, chronic lung disease, and malignant disease. Only 15% of patients were receiving osteoporosis treatment. According to nutritional status, the median CONUT score was 4 (2-6). Of the total 134 patients, 25 (15%), 56 (42%), 46 (34%) and 7 patients (5%) were classified as normal, light, moderate, and severe, respectively (Fig 2). 109 patients (81%) were malnourished. The mean GNRI and PNI was 94.8 ± 14.8 and 39.6 ± 6.5, respectively. Patients were divided into two groups according to the optimal CONUT value of 4 by the ROC curve to predict a decline in ambulatory ability. The CONUT-low group included patients with a CONUT score of 3 or less (*n = *58), and the CONUT-high group included patients with a CONUT score of 4 or more (*n = *76). From a post hoc power analysis, total sample size of 58 in the CONUT low group and 76 in the CONUT high group in the present study could achieve an adequate power 1-β 0.98 with an  α of 0.05.

**Table 2:** Differences in clinical characteristics and radiographic measurement

		CONUT		
	All patients (*n* = 134)	Low ( ≤ 3) *n* = 58	High ( ≥ 4) *n* = 76	*P*-value
Age, years W	83.5 ± 7.6	81.9 ± 7.4	84.7 ± 7.7	0.042*
Sex (n, % female)	89 (66)	42 (72)	47 (62)	0.20
BMI (kg/m^2^)	20.8 ± 4.3	22.7 ± 5.0	19.3 ± 3.7	$<$ 0.001*
Comorbidities (%)	112 (84)	46 (79)	66 (87)	0.25
Hypertension	60 (45)	31 (53)	29 (38)	0.078
Diabetes	25 (19)	9 (16)	16 (21)	0.41
Cardiovascular disease	23 (17)	9 (16)	14 (18)	0.66
Dementia	23 (17)	8 (14)	15 (20)	0.36
Cerebrovascular disease	10 (7)	3 (5)	7 (9)	0.37
Malignant disease	9 (7)	3 (5)	6 (8)	0.53
Chronic lung disease	8 (6)	3 (5)	5(7)	0.73
Osteoporosis treatment (%)	20 (15)	11 (19)	9 (12)	0.25
Hb (g/dl)	11.67 ± 1.92	12.57 ± 1.67	10.96 ± 1.82	$<$ 0.001*
Albumin (g/dl)	3.38 ± 0.57	3.80 ± 0.29	3.05 ± 0.52	$<$ 0.001*
Total lymphocyte cells (/mm^3^)	1183.7 ± 538.2	1394.8 ± 561.9	1022.7 ± 461.5	$<$ 0.001*
Total cholesterol (mg/dl)	174.9 ± 41.3	199.9 ± 37.2	155.8 ± 33.4	$<$ 0.001*
Level of AOVF, n (%)				
T10-	21 (16)	9 (16)	12 (16)	0.97
L1-T11	66 (49)	27 (47)	39 (51)	0.58
L2-5	60 (45)	25 (43)	35 (46)	0.73
Multiple AOVFs, n (%)	21 (16)	5 (9)	16 (21)	0.048*
Prevalent OVFs, n (%)	58 (43)	20 (34)	38 (50)	0.071
Lumbar Lordosis (°)	35.6 ± 14.2	36.5 ± 14.6	35.1 ± 13.9	0.54
BMD (g/cm^2^)	0.81 ± 0.14	0.83 ± 0.17	0.77 ± 0.084	0.61

*CONUT*, Controlling Nutritional Status, *BMI*, body mass index, *Hb*, hemoglobin, *AOVFs*, acute osteoporotic vertebral fractures, OVFs, osteoporotic vertebral fractures, *BMD*, bone mineral density

* *P*-value $<$ 0.05 statistically significant difference

### Comparison of patients between the CONUT-low group and the CONUT-high group

The CONUT-high group was significantly older and had significantly lower BMI and Hb than the CONUT-low group. There were no significant differences in sex, comorbidities and osteoporosis treatment (Table 2). Radiographic measurements showed that the presence of multiple AOVFs was significantly more common in the CONUT-high group than in the CONUT-low group (21% vs 9% *P* = 0.048). On the other hand, there was no significant difference in BMD between the CONUT-high and CONUT-low groups (Table 2). In terms of clinical outcome, although there was no significant difference in length of hospital stay, the CONUT-high group had a significantly higher rate of decline in ambulatory ability (67% vs. 33% *P* < 0.001), a significantly higher rate of complications during hospitalization (39% vs. 21% *P* = 0.019), and a significantly higher rate of discharge to nursing home (42% vs. 14% *P* < 0.001) than the CONUT-low group (Table 3).

**Table 3:** Comparison of clinical outcomes between CONUT score low and high group

		CONUT		
	All patients (*n* = 134)	Low ( ≤ 3) *n* = 58	High ( ≥ 4) *n* = 76	*P*-value
Decline in ambulatory ability (%)	70 (52)	19 (33)	51 (67)	$<$ 0.001*
Length of stay in hospital, day	39.5 ± 25.4	37.2 ± 24.5	41.4 ± 26.2	0.33
Discharge to nursing home (%)	40 (30)	8 (14)	32 (42)	$<$ 0.001*
Complications during hospitalization (%)	42 (31)	12 (21)	30 (39)	0.019*
Delirium	21 (16)	6 (10)	15 (20)	0.13
Pneumonia	10 (7)	3 (5)	7 (9)	0.37
Urinary tract infection	7 (5)	3 (5)	4 (5)	0.98

CONUT, Controlling Nutritional Status

* *P*-value $<$ 0.05 statistically significant difference

### Risk factor analysis associated with decline in ambulatory ability

In univariate analysis, the CONUT-high group ( ≥ 4) was associated with the decline in ambulatory ability (OR 4.18, 95% CI 2.02-8.67, *P* < 0.001) (Table 4). Osteoporosis treatment, complications during hospitalization, and multiple AOVFs were identified as cofactors associated with decreased ambulatory ability. The multivariable logistic regression analysis revealed that the CONUT score ( ≥ 4) was the independent risk factor associated with the decline in patient ambulatory ability (OR 3.44, 95% CI 1.61-7.37, *P* = 0.0014) (Table 4).

**Table 4:** Univariate and multivariate analysis of factors associated between CONUT score and decline in ambulatory ability

	Univariate analysis			Multivariate analysis		
	OR	95% CI	*P*-value	OR	95% CI	*P*-value
Age, years	2.49	0.38–16.3	0.345			
Sex, female	1.60	0.77–3.32	0.20			
BMI	0.37	0.05–2.52	0.30			
Comorbidities	1.11	0.45–2.78	0.82			
Osteoporosis treatment	0.33	0.12–0.93	0.030*	0.42	0.14–1.25	0.68
Complications	2.38	1.11–5.10	0.023*	1.86	0.82–4.22	0.12
Hb (g/dl)	0.28	0.03–2.31	0.23			
Multiple AOVFs	3.50	1.20–10.2	0.014*	2.61	0.85–8.02	0.14
Prevalent OVFs	1.09	0.55–2.16	0.81			
Lumbar lordosis	2.13	0.31–14.5	0.44			
BMD	0.56	0.11–2.74	0.48			
CONUT score $\ge$ 4	4.18	2.02–8.67	$<$ 0.001*	3.44	1.61–7.37	0.0014*

*CONUT*, Controlling Nutritional Status, *BMI*, body mass index, *Hb*, hemoglobin, *AOVFs*, acute osteoporotic vertebral fractures, *OVFs*, osteoporotic vertebral fractures, *BMD*, bone mineral density, *OR*, odds ratio, *CI*, confidence interval* *P*-value < 0.05 statistically significant difference

### Comparison of performance with CONUT score, GNRI and PNI to predict ambulatory ability

**Fig. 3:**
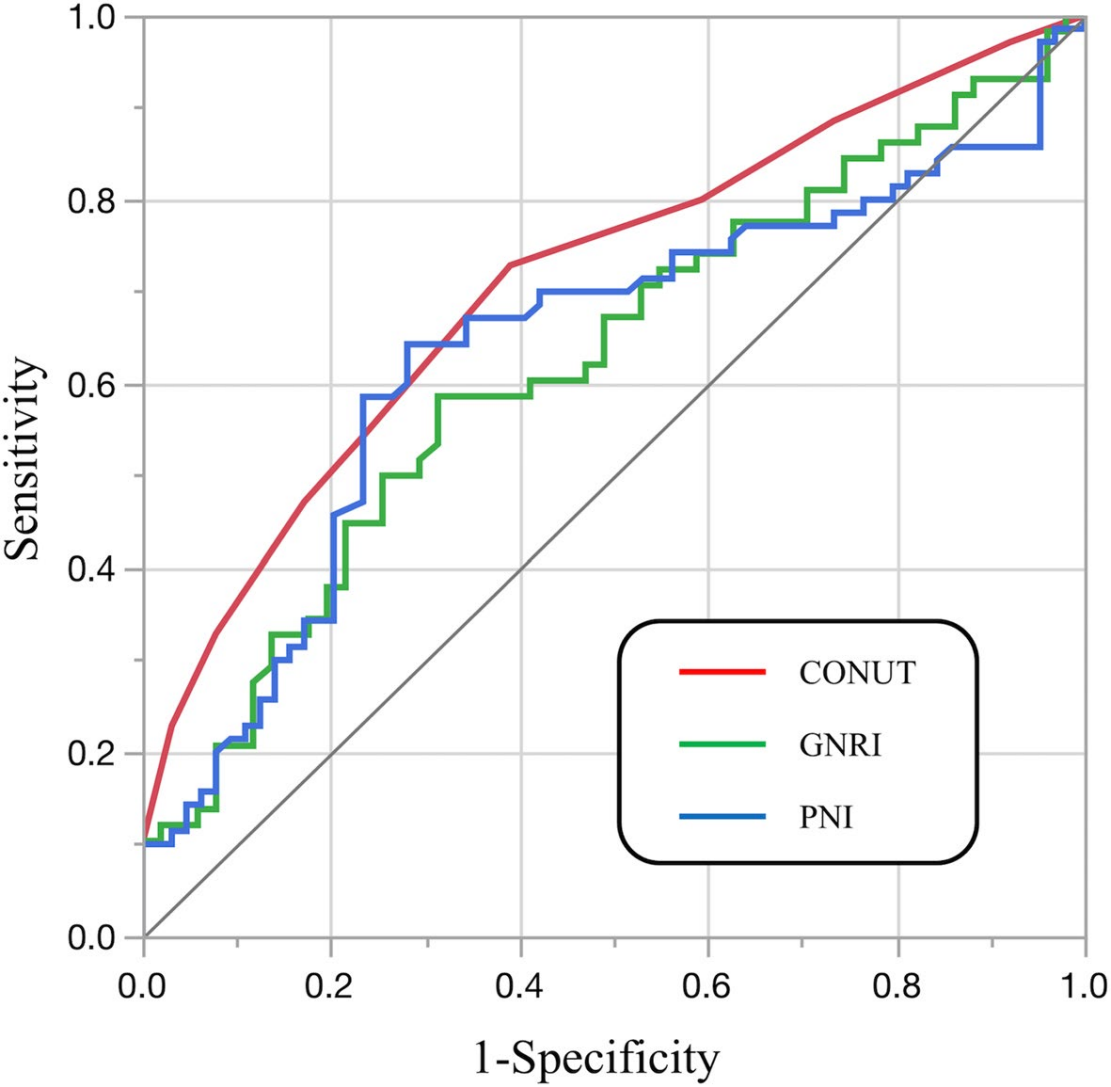
Comparison of receiver operating characteristic (ROC) curve for the decline in ambulatory ability. CONUT, Controlling Nutritional Status; GNRI, Geriatric Nutritional Risk Index; PNI, prognostic nutritional index

Finally, ROC curves for CONUT score, GNRI, and PNI were plotted to compare the prognostic value of the decline in ambulatory ability (Fig. 3). The optimal cutoff values for CONUT score, GNRI, and PNI for ambulatory ability were 4 (sensitivity = 72.9% and specificity = 61.0%, *P* < 0.001), 92.9 (sensitivity = 58.6 and specificity = 68.6%, *P* = 0.025), and 39.6 (sensitivity = 64.3% and specificity = 71.7%, *P* = 0.0077), respectively. The CONUT score performed significantly better than GNRI (AUC 0.724 vs 0.624, *P* = 0.021) and PNI (AUC 0.724 vs 0.624, *P* <  0.001) in predicting a decline in ambulatory ability. On the other hand, there were no significant differences in performance between GNRI and PNI (AUC 0.624 vs 0.639, *P* = 0.66) (Table 5).

**Table 5:** Comparing the areas under ROC curves of nutritional assessment tools for decline in ambulatory ability

Variables	AUC	95% CI	*P*-value
CONUT	0.724	0.631-0.800	$<$ 0.001*
GNRI	0.624	0.515–0.723	0.025 *
PNI	0.636	0.536–0.727	0.0077 *
Variables	Differences between areas	95% CI	*P*-value
CONUT - GNRI	0.0993	0.0148–0.184	0.021*
CONUT - PNI	0.0873	0.0227–0.152	< 0.001*
GNRI - PNI	-0.012	-0.066-0.0417	0.66

*ROC*, Receiver operating characteristic, *AUC*, area under the curve, *CONUT*, Controlling Nutritional Status, *GNRI*, Geriatric Nutritional Risk Index, *PNI*, prognostic nutritional index

* *P*-value $<$ 0.05 statistically significant difference

## Discussion

In this study, 81% of hospitalized patients with AOVF were malnourished. The CONUT-high group (malnourished patients) had more multiple AOVFs, more complications during hospitalization, and a higher rate of decline in ambulatory ability. The results showed that CONUT score is an independent risk factor associated with reduced ambulatory ability and had better performance in predicting the decline of ambulatory ability than GNRI and PNI.

The prevalence of malnutrition in OVF has been reported to be 36.4−61% [21, 22]. Nagai et al have investigated the nutritional status of OVF patients using GNRI and reported that 36.4% of patients with OVF were malnourished and that GNRI had a positive impact on ADL [21]. However, multiple AOVFs and prevalent OVFs were excluded from this study, and MRI was not used to diagnose OVF. These may have influenced the nutritional status and clinical outcome. On the other hand, 81% of patients enrolled in the study were malnourished. There were more malnourished patients compared to previous reports. The reason for this may be because the cohort focused on hospitalized patients with AOVFs and included patients with multiple AOVFs. Nutritional status has been reported to be associated with osteoporosis and osteoporotic fractures. Previous studies have reported that malnourished patients were 2.09 times more likely to develop osteoporosis and 1.28 times more likely to experience fracture [6, 23]. In this study, the CONUT-high group had significantly more multiple AOVFs than the CONUT-low group. However, there was no significant difference in the BMD between the CONUT-low and CONUT-high groups. The discrepancy may be due in part to the inaccuracy of DXA due to degenerative changes in the vertebrae and the effects of OVF.

In terms of association between nutritional status and functional prognosis, some studies have reported that nutritional status is a risk factor for ADL decline in the elderly. Malnutrition was reported in 30-50% of geriatric rehabilitation patients and 87.6% of patients admitted to acute care hospitals, and in both cases, patients’ nutritional status was significantly associated with lower ADL [24, 25]. Similar results in OVF patients were also reported. Previous retrospective studies have shown that the nutritional status of OVF patients evaluated with nutritional assessment tools such as GNRI and Mini Nutritional Assessment (MNA) was associated with the Barthel Index [21, 22]. In our series, 67% of patients in the CONUT-high ( ≥ 4) group experienced a decline in ambulatory ability, and CONUT score was an independent prognostic factor for decreased ambulatory ability upon discharge. Malnutrition leads to decreased muscle mass and body cell mass, resulting in sarcopenia and reduced physical function [26]. Furthermore, malnutrition has been reported to be a risk factor for osteoporosis and falling in patients with OVF [6], and subsequent fractures and further ADL decline are a concern. Our results suggest that nutritional status may affect functional prognosis of hospitalized patients with AOVF.

In addition to CONUT score, GNRI, and PNI, various nutrition assessment tools such as SGA and MNA have been validated [27, 28]. However, the ideal tool to evaluate nutritional status in elderly patients is controversial. It is impossible to calculate SGA and MNA when certain examinations and medical interviews are not conducted by healthcare professional. In addition, SGA has also been reported to have low inter-rater reliability (13%), suggesting that assessment tools such as SGA and MNA may be biased [29]. By contrast, ODA such as CONUT score, GNRI, and PNI are automatically calculated using objective values. Therefore, there is no need to consider inter-rater reliability. Because ODA does not require a medical interview, it is possible to assess the nutritional status of patients with dementia, which is often comorbid with AOVF in elderly patients. CONUT scores of patients with heart failure [18] and stroke [30] have been reported to be associated with functional prognosis. In a previous prospective study, Kojima et al reported that the CONUT score of patients with heart failure was an independent prognostic factor for the Barthel Index, however GNRI, PNI, and MNA had no relationship with the Barthel Index [18]. In our series, AUC of CONUT score, GNRI, and PNI for predicting the decline in ambulatory ability were 0.724, 0.624 and 0.636, respectively, and the CONUT score performed significantly better at predicting the ambulatory ability of hospitalized elderly patients with AOVFs. CONUT score includes total cholesterol in the parameter, unlike GNRI and PNI. Low cholesterol levels have been considered a reflection of protein energy malnutrition leading to low lipoprotein and ultimately sarcopenia and loss of muscle mass [31]. Therefore, the CONUT score is more likely to reflect functional prognosis than GNRI or PNI.

In terms of complications during hospitalization, Yagi et al showed that the CONUT score was associated with postoperative complications after hip fracture surgery [32]. Although this study focused on conservative AOVF treatment, the CONUT-high group had significantly more complications than the CONUT-low group, which is consistent with Yagi’s suggestion.

There are some limitations to this study. First, this study included only hospitalized patients with significant disability due to severe low back pain, and the study cohort may be biased toward patients with low ADLs. Therefore, our findings may not be applicable to the entire AOVF patient population. Second, there were insufficient data on the treatment of osteoporosis in this study, and details on the type of braces and physical therapy used under conservative treatment. Third, we did not have enough data regarding living environment, such as number of family members, whether or not people were living together, the presence or absence of stairs in the home, which could affect clinical outcomes. Fourth, we did not assess sarcopenia and muscle loss, and could not evaluate their effect on nutritional status and clinical outcome. Fifth, the present study excluded 51 of 192 cases due to lack of data. This may have influenced our results. Finally, the design of present study is monocentric and retrospective observational study. Systematic errors (e.g., selection bias) are present to some degree in the results and our findings may not be generalized. Therefore, prospective muti-center study or prospective intervention studies are desirable in the future.

In conclusion, the current study demonstrated that 81% of hospitalized elderly patients with AOVFs were malnourished based on CONUT score. The CONUT score was an independent risk factor associated with the decline in ambulatory ability upon discharge and had better predictive performance for decreased ambulatory ability than other nutritional assessment tools. Our findings suggest that nutritional assessment using CONUT score may be important for improving functional prognosis after AOVFs. Further research is needed on the impact of nutritional support interventions on elderly patients with AOVFs.

## Data Availability

The datasets used and/or analyzed during the current study are available from the corresponding author on reasonable request.
